# Measuring quality-of-care in the context of sustainable development goal 3: a call for papers

**DOI:** 10.2471/BLT.16.170605

**Published:** 2016-03-01

**Authors:** Yoko Akachi, Finn Tarp, Edward Kelley, Tony Addison, Margaret E Kruk

**Affiliations:** a UNU-WIDER, Katajanokanlaituri 6B, FI-00160, Helsinki, Finland.; b Department of Service Delivery and Safety, World Health Organization, Geneva, Switzerland.; c Department of Global Health and Population, Harvard TH Chan School of Public Health, Boston, United States of America.

Global and national efforts to meet the health Millennium Development Goals (MDGs) expanded basic health-care coverage to tackle infectious diseases and child mortality. These efforts met with some success. However, a focus solely on health-care coverage is unlikely to be sufficient to meet sustainable development goal (SDG) 3 to “ensure healthy lives and promote well-being for all at all ages”. To reach this goal, we need to address a critical issue: the quality of health care.

High-income countries already invest considerable resources in measuring the level and variation in health-care quality and associations with health outcomes.^1^^,^^2^ There is much less emphasis on quality measurement in low- and middle-income countries, although individual studies suggest that poor quality is limiting health gains.^3^^–^^6^ A study using data from the World Health Organization (WHO) Multicountry Survey on Maternal and Newborn Health^3^ found that high coverage of essential interventions was not associated with reduced maternal mortality in facilities, concluding that the coverage of interventions must be matched with overall improvements in quality-of-care. Other recent studies from India, Malawi and Rwanda showed that the higher rates of institutional deliveries and better access to antenatal care were not accompanied by reductions in maternal and newborn mortality. These studies concluded that poor quality of clinical services is likely to be a factor.^4^^–^^6^ Poor quality-of-care has also undermined the control of diseases such as malaria and human immunodeficiency virus (HIV) infections,^7^^,^^8^ and is common in the treatment of noncommunicable diseases, surgical conditions, and mental health conditions.^9^^,^^10^ These findings suggest that to address new health priorities in the SDGs, quality must be measured and determinants of quality performance identified. Such measures are especially critical given the large scope and increased complexity of health services required.

The universal health coverage (UHC) target of the health SDG stipulates that everyone can obtain essential health services at high quality without suffering financial hardship, yet quality has not been widely tracked.^11^ There is no benefit to UHC if people are unwilling to use services due to the poor quality of the services for which they are financially covered. Even if people are accessing services, poor quality will undermine health outcomes, reducing the value of UHC. Finally, high-quality health services attract the public support that contributes to governments providing sustained financing.

One reason for the lack of data on quality of health-care services in low- and middle-income countries may be the past emphasis on coverage rather than the challenge of providing high-quality services. The quality measures that exist are not sufficiently validated. Measurements of quality are not done consistently and therefore it is often not possible to compare between settings. The best measures, such as clinical observations, are expensive to collect and thus difficult to use at scale and over time. New, validated and feasible measures are therefore needed for each dimension: infrastructure and staffing (equipment, drugs and vaccines, health workers), technical quality (competence of health-care providers, compliance with good practice recommendations), and patient experience (convenience, dignity, communication).^12^

Against this background, the *Bulletin of the World Health Organization* will publish a theme issue on quality-of-care in the era of SDGs. This theme issue will include original research articles on quality-of-care in low- and middle-income countries. The selection of this research will prioritize rigorous methods, generalizability across contexts, and salience of findings for policy. We welcome papers for all sections of the *Bulletin,* around two themes: measurement of health-care quality and associations between quality improvement measures and health outcomes. What are candidate indicators for measuring quality-of-care in low- and middle-income countries? How can we measure quality-corrected population coverage of interventions and services to permit comparisons within and between countries? We also welcome analyses of the links between poor quality and health outcomes, the sources of variance in quality-of-care, the equity dimensions of health-care quality, and the drivers of service quality improvement.

The deadline for submission is 31 May 2016. Manuscripts should be submitted in accordance with the *Bulletin´s* *guidelines for contributors* (http://www.who.int/bulletin/contributors), and the cover letter should mention this call for papers. All submissions will be peer-reviewed.
